# Protecting the Heart in Motion: The Role of Physical Activity and Cardiorespiratory Fitness in Preventing Sudden Cardiac Death

**DOI:** 10.1177/11795468251391010

**Published:** 2025-12-18

**Authors:** Setor K. Kunutsor, Khushmanjot Kaur, Jari A. Laukkanen

**Affiliations:** 1 Department of Internal Medicine, Max Rady College of Medicine, Rady Faculty of Health Sciences, University of Manitoba, Winnipeg, Canada; 2 I.H. Asper Institute, St. Boniface Hospital, Winnipeg, MB; 3 Department of Physiology and Pathophysiology, Rady Faculty of Health Sciences, University of Manitoba, MB, Canada; 4 Department of Medicine, University of Eastern Finland, Kuopio, Finland; 5 Department of Medicine, Wellbeing Services County of Central Finland, Jyväskylä, Finland

**Keywords:** sudden cardiac death, physical activity, cardiorespiratory fitness, prevention, cardiovascular risk, lifestyle

## Abstract

Sudden cardiac death (SCD) remains one of the most devastating manifestations of cardiovascular disease. While traditional risk stratification has focused on structural heart disease and electrophysiological markers, growing evidence suggests that modifiable lifestyle factors—particularly physical activity (PA) and cardiorespiratory fitness (CRF)—play a critical role in mitigating the risk of SCD. This narrative review synthesizes evidence on the associations between PA, CRF, and SCD risk. It explores potential biological mechanisms underlying these relationships, identifies key gaps in the literature, and discusses the clinical and public health implications. A substantial body of prospective cohort studies and meta-analyses demonstrates a strong, inverse, and dose-dependent association between both PA and CRF and the risk of SCD. Engaging in ⩾4 hours/week of moderate-to-vigorous PA or achieving CRF levels of ⩾8 to 10 METs is associated with 40% to 50% reductions in SCD risk. CRF also modifies the risk conferred by traditional cardiovascular risk factors such as hypertension, diabetes, and systemic inflammation. Proposed mechanisms include favorable modulation of cardiovascular risk profiles, improved autonomic regulation, anti-arrhythmic and anti-ischemic effects, and enhanced myocardial function. However, evidence gaps persist regarding causal inference (absence of Mendelian randomization studies), optimal PA and CRF thresholds, sex- and age-specific effects, and interactions with other risk factors. PA and CRF are powerful, modifiable predictors of SCD and should be integrated into preventive strategies and routine clinical assessments. Targeted interventions to increase PA and improve CRF, especially among underrepresented and high-risk groups, offer an important opportunity to reduce the burden of SCD globally.

## Introduction

Sudden cardiac death (SCD) is a catastrophic and often unpredictable event that accounts for a substantial proportion of cardiovascular mortality worldwide.^1^ Defined as an unexpected death due to cardiac causes occurring within a short time period—typically within 1 hour of symptom onset—SCD affects both individuals with known cardiovascular disease (CVD) and those without prior warning signs.^2^ Globally, it is estimated that SCD is responsible for 15% to 20% of all deaths,^1^ with annual incidence rates ranging from 40 to 100 per 100,000 population depending on geographic region, age, and sex.^3^ Although coronary artery disease (CAD) is the most common underlying pathology in adults,^1,2^ SCD can also occur in structurally normal hearts, particularly in younger individuals and athletes.^2^ Traditional risk factors for SCD include left ventricular dysfunction, ischemic heart disease, arrhythmogenic conditions, and genetic syndromes;^2,4^ however, these account for only a subset of total events. Increasing attention is being directed toward modifiable risk factors—particularly those related to lifestyle—given their potential for wide-scale prevention.

Lifestyle factors such as poor diet, tobacco use, physical inactivity, and excess alcohol consumption are well-established contributors to cardiovascular morbidity and mortality.^4,5^ Physical inactivity in particular has been strongly associated with adverse cardiometabolic profiles and elevated risk of non-communicable diseases including coronary heart disease (CHD), stroke, type 2 diabetes (T2D), and several cancers.^6^ Regular physical activity (PA), on the other hand, exerts wide-ranging protective effects across multiple organ systems, conferring both vascular ^7–16^ and non-vascular benefits.^9,17–19^ These include improved lipid profiles, blood pressure regulation, enhanced glucose metabolism, and anti-inflammatory effects, among others.^15,20–23^ The benefits of PA extend beyond traditional cardiovascular outcomes to also include improved psychological well-being, cognitive function, and overall longevity.^24^ Closely related to PA is cardiorespiratory fitness (CRF)—a measurable and objective indicator of the body’s capacity to transport and utilize oxygen during sustained exercise.^25,26^ CRF reflects both intrinsic physiological capacity and the cumulative effects of habitual PA. It serves as a robust marker of cardiovascular and metabolic health and has emerged as a powerful, independent predictor of a wide range of adverse outcomes, including all-cause mortality, cardiovascular events, and heart failure.^16,25,27–35^ The associations between CRF and cardiovascular risk have consistently been shown to be inverse, graded, and independent of traditional risk factors.^25,27–30,36^ These relationships are also remarkably consistent across diverse demographic groups, including different ages, sexes, and racial or ethnic populations.^37,38^ In light of the growing body of evidence supporting its prognostic value, the American Heart Association issued a Scientific Statement in 2016 recommending that CRF be regarded as a clinical vital sign and assessed routinely alongside other established cardiovascular risk indicators.^25,26^ Nonetheless, CRF remains absent from most standard cardiovascular risk prediction tools, highlighting a critical disconnect between its clinical relevance and practical implementation.

Despite the strong evidence base linking PA and CRF to cardiovascular outcomes broadly, their specific roles in the prevention of SCD remain underexplored and underemphasized in current prevention guidelines. While observational studies have shown inverse associations between these factors and SCD risk,^39,40^ further understanding of the mechanisms, dose-response relationships, and population-specific effects is essential for clinical translation. Additionally, integrating CRF and PA into predictive algorithms and preventive strategies for SCD could improve risk stratification, particularly in individuals without overt CVD.

This narrative review aims to (i) summarize existing evidence on the associations—both observational and causal—of PA and CRF with the risk of SCD, (ii) explore potential biological mechanisms through which PA and CRF may influence SCD risk, (iii) assess differences in impact across population subgroups (e.g., age, sex, presence of underlying disease), and (iv) identify gaps in the current evidence base and propose directions for future research. By synthesizing current knowledge and highlighting areas for advancement, this review seeks to inform both clinical practice and population health strategies aimed at reducing the burden of SCD through lifestyle-based prevention.

## Methods

A comprehensive literature search was performed in MEDLINE and Embase up to May 2025, targeting both observational studies (including prospective cohort, nested case-control, case-cohort, and retrospective cohort designs) and interventional trials. Particular emphasis was placed on identifying relevant systematic reviews and meta-analyses, where available, in line with the principles of evidence hierarchy.^41^ The search strategy involved a combination of terms related to PA and CRF (e.g., “*physical activity*,” *“exercise*,” *“aerobic exercise,” “cardiorespiratory fitness,” “CRF,” “aerobic fitness,” “cardiovascular fitness*,” *“aerobic capacity,” “cardio fitness,” “physical fitness,” “maximal oxygen uptake,” “VO2max,” “peak oxygen consumption,” “VO2peak”*) and terms relevant to SCD (e.g., “*sudden cardiac death,” “SCD,” “sudden unexpected cardiac death,” “SUCD,” “sudden death,” “sudden cardiac arrest,” “cardiac arrest”*). The search was restricted to studies involving adult human populations, published in English, and excluded cross-sectional designs due to their inability to establish temporal relationships. We extracted and reported risk estimates (effect estimates (ES), relative risks (RR), odds ratios (ORs), hazard ratios (HRs), mean differences and standardized mean differences (SMDs)) for associations demonstrating significant effects. Fully adjusted risk estimates were reported. Because the review focused on the general population, studies conducted exclusively in athletic populations or assessing competitive or endurance sports were not included. Individuals engaged in high-level athletic or endurance activities represent a physiologically distinct demographic with long-standing adaptations to intensive training, making them not representative of the general population. Nonetheless, we included a dedicated section on “Special Populations” to explore the unique considerations surrounding SCD risk among athletes, marathon runners, those involved in endurance sports as well as those with pre-existing CVD. We only included studies that used direct measures of CRF and not other parameters derived from CRF such as peak exercise oxygen pulse (peak oxygen uptake/heart rate), cardiorespiratory optimal point, percentage of age-predicted CRF, hemodynamic gain index, etc. Finally, to assess potential causal associations of PA and CRF with SCD, a separate search was conducted for Mendelian randomization (MR) studies evaluating genetic proxies for PA or CRF in relation to SCD outcomes.

## Definitions

To provide conceptual clarity and avoid ambiguity in the discussion that follows, it is essential to distinguish between the related but distinct terms: PA, exercise, and CRF—all of which are central to this review. PA refers broadly to any bodily movement produced by skeletal muscles that results in energy expenditure above resting levels.^42,43^ This includes a range of activities such as occupational tasks, domestic chores, commuting, and recreational pursuits.^44^ In contrast, exercise is a structured and purposeful subset of PA that is planned and repetitive, with the specific aim of improving or maintaining one or more components of physical fitness.^43^ Exercise can be further classified into types such as aerobic (e.g., brisk walking, cycling), resistance (e.g., weightlifting), and high-intensity interval training (HIIT).^45^ CRF, distinct from PA and exercise, is a measurable physiological attribute that reflects the efficiency of the cardiovascular and respiratory systems in delivering oxygen to skeletal muscle during sustained physical effort.^25,43^ CRF is influenced by both genetic factors and engagement in regular aerobic activity and is commonly considered a functional outcome of habitual PA and exercise training.^25,43^ CRF is also known as aerobic capacity, and is often quantified using maximum oxygen intake (VO₂max) or peak oxygen uptake (VO₂peak), depending on how the assessment is conducted.^26^

There are several approaches to assessing PA and CRF in both observational and interventional research. PA is typically measured through self-report instruments such as questionnaires or diaries, though objective methods like accelerometry and pedometry are increasingly used for greater accuracy. CRF assessment varies by study design and setting. The gold standard is cardiopulmonary exercise testing (CPX) using a treadmill or cycle ergometer, which provides direct measurements of VO₂max.^25^ VO₂max is defined as the maximum rate of oxygen consumption attained during maximal exercise, plateauing even with increased workload—indicating the individual's peak aerobic capacity.^46,47^ When a true plateau is not observed, the highest VO₂ value attained is reported as VO₂peak.^48^ However, in large epidemiological studies and clinical practice where direct testing is not feasible, estimation from exercise tests or attained workload and non-exercise-based estimation equations are often used.^49,50^ Non-exercise-based estimation equations rely on surrogate measures such as self-reported PA, resting heart rate, body mass index (BMI), and age to provide a reasonably accurate and cost-effective proxy for CRF.^25,26^ While practical for population-level research, these estimations have limitations, including potential under- or overestimation at the extremes of the fitness spectrum and variability based on the algorithms and input measures used. Not all prediction equations are universally applicable across diverse populations due to methodological heterogeneity.^25,26^ CRF is typically expressed in mL/kg/min or metabolic equivalents (METs)—with one MET defined as the resting energy expenditure, equivalent to approximately 3.5 mL/kg/min in the average adult.^51^

## Physical Activity and Sudden Cardiac Death

Several observational studies over the past decades have consistently demonstrated an inverse association between PA and the risk of SCD, suggesting a protective effect across diverse populations and study settings.

In one of the earliest prospective investigations, Wannamethee et al. (1995) examined a cohort of men from 24 British towns followed for 8 years and found that those engaging in moderately vigorous to vigorous PA had a significantly lower risk of SCD compared to their sedentary counterparts (RR = 0.4, 95% CI: 0.2-0.7).^52^ Similarly, in the Physicians’ Health Study, Albert et al. (2000) used a nested case-crossover design to evaluate the transient and habitual effects of vigorous PA. Although the relative risk of SCD during and immediately following an episode of vigorous exertion was markedly elevated (RR = 16.9, 95% CI: 10.5-27.0), habitual vigorous exercise was shown to substantially attenuate this risk.^53^ In 2006, Whang and colleagues assessed about 70 000 women from the Nurses’ Health Study (NHS) and demonstrated a strong inverse relationship between weekly exercise duration and SCD risk. Compared to inactive women, those engaging in ⩾4 hours/week of moderate-to-vigorous exercise had a 49% lower risk (HR = 0.51, 95% CI: 0.47-0.56).^54^ Extending this evidence, Chiuve et al. (2011), also using the NHS, reported that women exercising ⩾30 minutes/day had a lower risk of SCD (RR = 0.67, 95% CI: 0.50-0.90), with a stronger reduction observed in those exercising ⩾6.0 vs ⩽1.0 hours/week (RR = 0.47, 95% CI: 0.30-0.72).^55^ In the FINRISK and Health 2000 cohort studies, Lahtinen et al. (2012) conducted an individual participant data meta-analysis and found that moderate-to-high levels of leisure-time PA (LTPA) were associated with a significantly reduced risk of SCD (HR = 0.51, 95% CI: 0.43-0.62) compared to low LTPA.^56^ The most comprehensive summary to date was provided by Aune et al. (2020) in a meta-analysis of eight prospective cohort studies. They reported that individuals with the highest levels of PA had a 48% lower risk of SCD compared to those with the lowest levels (RR = 0.52, 95% CI: 0.45-0.60). Dose-response analyses indicated that each 20 MET-hours/week increment in PA was associated with a 32% risk reduction, though no additional benefit was observed beyond 20 to 25 MET-hours/week. The association was consistent across sex and geographic regions.^39^ Using implantable devices, Li et al. (2020) examined a high-risk population and observed a non-linear inverse association between daily PA and cardiac death. The risk was halved at a PA level of 12.3% of daily activity (≈177 min/day), after which the benefit plateaued. Notably, older adults (⩾60 years) appeared to derive greater benefit.^57^ Wolthers et al. (2022) investigated out-of-hospital cardiac arrest (OHCA) events using Danish registry data and found that only 2.2% of OHCAs occurred during exercise. Importantly, exercise-related arrests had a substantially higher 30-day survival rate (57.7% vs 12.6%) compared to non-exercise-related events, with team sports offering the highest survival rates.^58^ Also in 2022, Jin and colleagues used the Korean National Health Screening Program database to quantify the dose-response association between moderate-to-vigorous PA and primary cardiac arrest. They observed a curvilinear association with maximal benefit at 2 to 3 times the guideline-recommended minimum (HR = 0.6, 95% CI: 0.4-0.8), with no indication of harm at very high PA levels.^59^ In a separate analysis of older adults (>75 years) at elevated risk of SCD, Li et al. (2022) demonstrated that individuals engaging in ⩽93 minutes/day of objectively measured PA had a significantly greater risk of cardiac death compared to those exceeding this threshold (HR = 3.35, 95% CI: 1.39-8.03).^60^ In an umbrella review of 55 meta-analyses, Tsartsalis et al. (2022) identified PA as a robust protective factor against SCD among numerous lifestyle and clinical exposures.^4^ Using accelerometry data from nearly 100 000 participants in the UK Biobank, Qiu and Xing (2023) showed that higher total PA, moderate PA, and vigorous PA were all independently associated with a lower risk of cardiac arrest. Minutes per week of light PA, moderate PA, and vigorous PA were defined as the time spent in 30 to 125 mg, 125 to 400 mg, and >400 mg intensity activity, respectively. Risk reductions per standard deviation increase in activity were 27% for total PA, 24% for moderate PA, and 20% for vigorous PA. The risk of cardiac arrest declined steeply with increasing PA volume, with the most notable risk reduction observed up to 360 minutes/week for moderate PA and 20 minutes/week for vigorous PA, beyond which the benefit plateaued. Protective effects were more pronounced in women than in men.^61^ Finally, a recent analysis from the Copenhagen City Heart Study by Empana et al. (2025) assessed cardiovascular health (CVH) metrics over a 10-year period and their association with SCD. Interestingly, neither baseline PA nor its change over time showed a significant association with SCD risk, in contrast to other CVH metrics.^62^

In summary, a substantial body of observational evidence demonstrates a consistent inverse association between PA and the risk of SCD, with optimal protection observed at moderate-to-vigorous PA levels. Across multiple cohorts, engaging in ⩾4 hours/week or ⩾30 minutes/day of moderate-to-vigorous activity was associated with 40 to 50% lower SCD risk, while dose–response data suggest that the greatest risk reduction occurs up to 20 to 25 MET-hours/week or 360 minutes/week of moderate PA and 20 minutes/week of vigorous PA, beyond which benefits tend to plateau. Although one recent study found no association between PA and SCD when assessed as part of overall cardiovascular health metrics, the weight of evidence supports PA as a key modifiable protective factor against SCD.

## Cardiorespiratory Fitness and Sudden Cardiac Death

A growing body of evidence has specifically linked higher CRF levels to a reduced risk of SCD, independent of traditional risk factors. In a landmark study from the Kuopio Ischemic Heart Disease (KIHD) cohort in Finland, Laukkanen et al (2010) followed 2368 men and found a significant inverse association between CRF (measured using a respiratory gas exchange analyzer) and SCD risk. Each 1-MET increase in CRF was associated with a 22% lower risk of SCD (RR = 0.78, 95% CI: 0.71-0.84). Notably, the inclusion of CRF improved risk prediction beyond conventional cardiovascular risk factors.^35^ Using data from the Aerobics Center Longitudinal Study (ACLS), Jiménez-Pavón et al. (2016) assessed SCD risk across categories of CRF measured via maximal treadmill testing. Compared to individuals with low CRF, those with moderate and high CRF had significantly lower risks of SCD (HR = 0.56, 95% CI: 0.35–0.90 and HR = 0.52, 95% CI: 0.30-0.92, respectively). Additionally, each 1-MET increase in CRF was associated with a 14% reduction in SCD risk (HR = 0.86, 95% CI: 0.77-0.96), with no significant differences in association by sex.^63^ In a systematic review and meta-analysis, Jiménez-Pavón et al. (2019) synthesized data from 11 prospective cohort studies examining CRF or CRF proxies in relation to SCD. Only two of these studies specifically evaluated direct CRF-SCD associations. A pooled analysis of these two studies found that each 1-MET increase in CRF was associated with a 19% reduction in SCD risk (HR = 0.81, 95% CI: 0.74-0.89).^40^ The Finnish Cardiovascular Study further contributed to the evidence base, where Hernesniemi and colleagues (2019) assessed CRF through maximal work level achieved during cycle ergometer testing. They found that a 1-standard deviation increase in CRF was associated with a significantly lower risk of SCD (HR = 0.69, 95% CI: 0.52-0.93), with similar associations observed in men and women.^64^ In a 2020 meta-analysis, Aune et al. incorporated two cohort studies evaluating CRF and SCD and found a strong inverse association: RR = 0.58 (95% CI: 0.41-0.81) when comparing the highest versus lowest CRF levels, and RR = 0.49 (95% CI: 0.33-0.73) per 5-MET increase in CRF.^39^

Collectively, findings from large-scale prospective cohort studies and meta-analyses provide consistent evidence that higher levels of CRF are associated with a substantially reduced risk of SCD. This protective association appears robust across different populations, measurement techniques, and sexes. The evidence demonstrates a strong, inverse, and dose-dependent association between CRF and the risk of SCD, independent of traditional cardiovascular risk factors. Across multiple cohort studies, each 1-MET increase in CRF was associated with a 14% to 22% lower risk of SCD, with the greatest protection observed in individuals achieving CRF levels of ⩾8 to 10 METs, where risk reductions exceeded 40% to 50%. These findings suggest that even modest improvements in CRF may confer meaningful reductions in SCD risk and highlight CRF as a critical and modifiable preventive target. Importantly, CRF may enhance risk prediction beyond traditional risk models.

## Evidence from Mendelian Randomization Studies

MR has emerged as a powerful methodological approach for evaluating potential causal relationships between modifiable exposures and health outcomes. By leveraging genetic variants—typically single nucleotide polymorphisms (SNPs)—as instrumental variables, MR helps to reduce the biases of confounding and reverse causality that often affect observational epidemiology.^65^ In the context of this review, MR offers a potentially valuable tool to investigate whether levels of PA and CRF are causally associated with the risk of SCD. Despite the biological plausibility and observational evidence linking higher levels of PA and CRF to lower SCD risk, our search identified no MR studies to date that have directly assessed the causal relationship between either CRF or PA and SCD. This gap likely reflects both the relative rarity and complexity of SCD as an endpoint, and challenges in constructing valid genetic instruments for CRF and PA that are robust enough to detect effects on relatively uncommon outcomes such as SCD. We found only one MR study that explored the causal associations of CRF with age-related diseases, using data from the UK Biobank.^66^ This study assessed the impact of genetically predicted CRF on several chronic conditions, including CVD, Alzheimer’s disease, and Parkinson’s disease. The authors reported no clear evidence of a causal relationship between CRF and CVD outcomes.^66^ Notably, other MR studies have explored the associations of genetically predicted CRF with conditions such as type 2 diabetes and various cancers, with mixed findings.^67,68^ However, the field of MR for CRF remains nascent, possibly due to the difficulty in identifying genetic variants that precisely reflect CRF levels in the general population. Most genome-wide association studies (GWAS) for CRF have relied on resting heart rate (RHR) as a proxy, given its inverse correlation with CRF and its responsiveness to aerobic exercise training.^69–71^ While useful, RHR is not a direct measure of fitness and introduces limitations when used as a surrogate in causal inference models.

Similarly, no MR studies have specifically examined the causal relationship between PA and SCD. Nonetheless, a growing body of MR research has evaluated the impact of PA on broader cardiovascular outcomes. Several studies have reported protective causal effects of genetically predicted vigorous or total PA on outcomes such as CAD and myocardial infarction,^72,73^ while others have found no causal associations.^74,75^

## Potential Pathways Underlying the Effects of PA and CRF on SCD in the General Population

**Figure 1.**
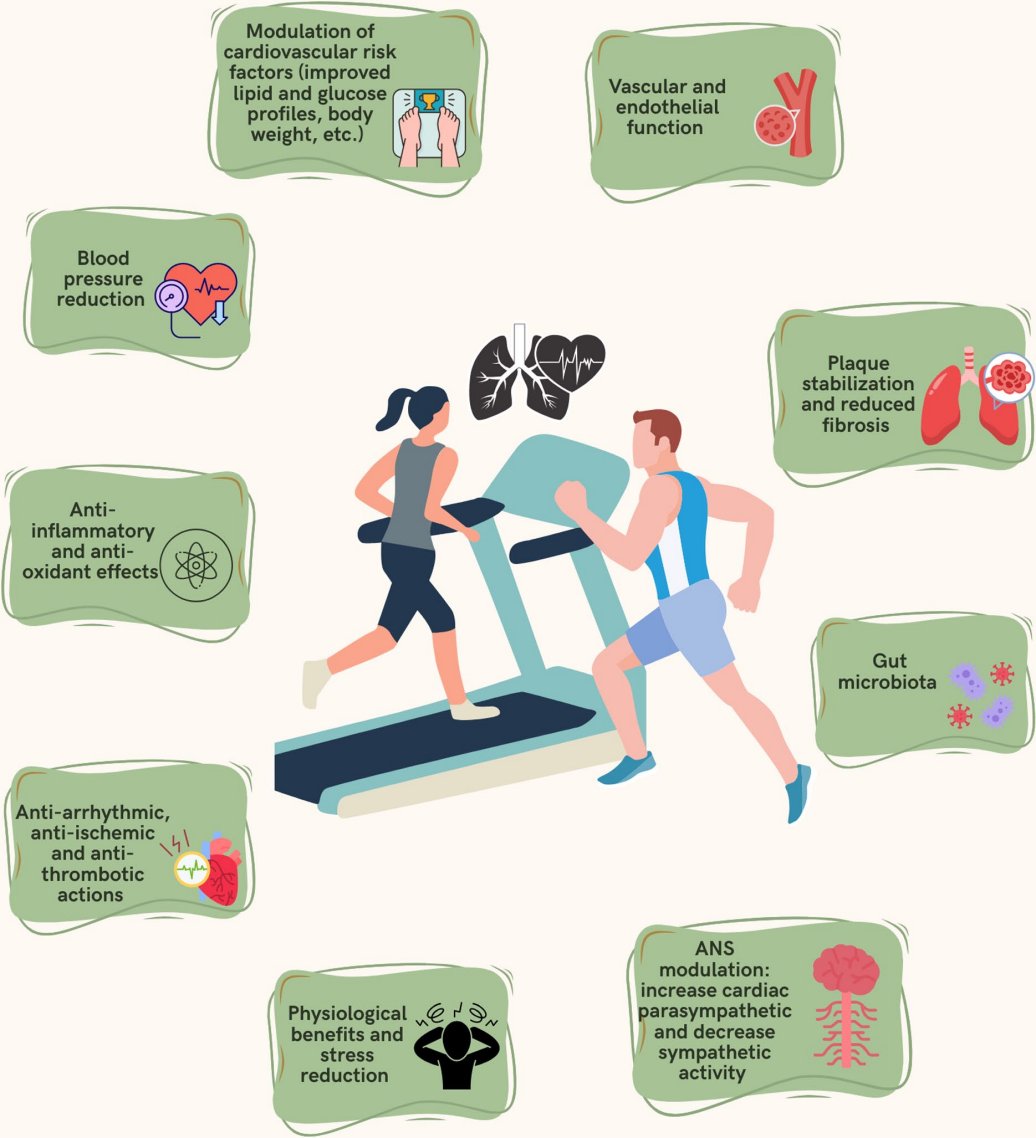
Proposed mechanistic pathways underlying the beneficial effects of PA and CRF on SCD risk. Abbreviations: ANS, autonomic nervous system; CRF, cardiorespiratory fitness; PA, physical activity; SCD, sudden cardiac death.

The protective effects of PA and CRF against SCD are increasingly supported by epidemiological evidence (Table 1). CRF, often viewed as a cumulative marker of habitual PA and exercise behavior,^76^ reflects both the integrative functional capacity of the cardiovascular, pulmonary, and musculoskeletal systems and the adaptive physiological changes induced by sustained physical exertion. The associations observed between higher CRF levels and reduced SCD risk are likely mediated through multiple, interconnected biological pathways, many of which are directly influenced by PA and exercise. As illustrated in Figure 1, PA and/or exercise initiates a cascade of favorable adaptations across several organ systems and physiological processes that may collectively reduce the risk of SCD:

*Modulation of traditional cardiovascular risk factors*: Regular PA improves lipid metabolism, enhances glucose regulation, reduces insulin resistance, and supports healthy body weight maintenance ^15,20–23^—factors that are independently associated with reduced cardiovascular morbidity and mortality. These effects collectively contribute to a reduction in atherosclerotic burden, a major substrate for SCD.
*Blood pressure reduction*: Exercise has well-established antihypertensive effects, reducing both systolic and diastolic blood pressure through improved vascular compliance, arterial function, and baroreflex sensitivity.^77^ Since hypertension is a known contributor to left ventricular hypertrophy and arrhythmogenic remodeling,^78^ lowering blood pressure may directly reduce SCD risk.
*Anti-inflammatory and anti-oxidant effects*: PA suppresses systemic inflammation by downregulating pro-inflammatory cytokines and enhancing anti-inflammatory mediators.^15,79,80^ It also reduces oxidative stress,^81^, thereby mitigating endothelial dysfunction and myocardial damage—both of which are implicated in arrhythmogenesis and SCD.
*Anti-arrhythmic, anti-ischemic, and anti-thrombotic Actions*: PA and high CRF levels may exert direct myocardial protection by decreasing myocardial oxygen demand, improving coronary perfusion, reducing platelet aggregation, and stabilizing myocardial electrical activity.^80^ These effects reduce susceptibility to lethal ventricular arrhythmias, the predominant mechanism underlying SCD.
*Autonomic nervous system (ANS) modulation*: Regular aerobic exercise leads to enhanced parasympathetic tone and attenuated sympathetic activity at rest, improving heart rate variability and reducing arrhythmic potential.^80,82–84^ This improved autonomic balance is particularly relevant given the role of sympathetic overactivation in SCD pathophysiology.
*Vascular and endothelial function*: Exercise promotes nitric oxide bioavailability and endothelial-dependent vasodilation,^85,86^ contributing to healthy vascular function and arterial elasticity, thereby reducing the risk of ischemic triggers for SCD.
*Plaque stabilization and reduced fibrosis*: PA may aid in stabilizing atherosclerotic plaques by promoting a favorable plaque phenotype (i.e., thicker fibrous cap, reduced necrotic core) and reducing matrix metalloproteinase activity.^87^ Additionally, it may attenuate myocardial fibrosis,^88^ preserving myocardial structure and electrical integrity.
*Psychological benefits and stress reduction*: Exercise enhances sleep quality, reduces anxiety, and modulates stress perception via both central nervous system mechanisms and peripheral autonomic modulation.^15,89–91^ These psychological benefits may reduce adrenergic surges that trigger arrhythmic events in vulnerable individuals.
*Gut microbiota and metabolic homeostasis*: Emerging evidence suggests that exercise positively modulates the gut microbiota,^80^ which in turn influences systemic inflammation, metabolic regulation, and cardiovascular risk—highlighting a novel indirect pathway for SCD prevention.^92^

Collectively, these mechanisms offer a plausible biological framework for understanding how sustained PA and higher CRF confer protection against SCD in the general population. Importantly, these effects are multi-systemic and synergistic, with many pathways reinforcing one another. While observational and mechanistic data are compelling, further work is needed to elucidate the relative contributions of these pathways, their temporal dynamics, and threshold effects in diverse populations.

**Table 1.** Key Studies and Meta-Analyses that Have Evaluated the Associations of Physical Activity and Cardiorespiratory Fitness with Sudden Cardiac Death.

Author, year of publication [reference]	Country	Study design	Assessment method for PA/CRF	Number of studies / Events / Participants	Follow-up, yrs	Results (Multivariable adjusted estimates)	Summary/Notes
Physical activity							
Wannamethee (1995)^52^	UK	Prospective cohort	Self-reported	Sex = Males Sample = 7735 Events = 117	8.0	RR (95% CI) of 0.4 (0.2-0.7) comparing moderately vigorous to vigorous PA vs none to occasional PA	Inverse association between PA and SCD
Albert (2000)^53^	USA	Prospective nested case-crossover design	Self-reported	Sex = Males Sample = 21,481 Events = 122	12.0	RR (95% CI) of 16.9 (10.5-27.0) associated with an episode of vigorous exertion; risk lowered with habitual vigorous exercise	Habitual vigorous exercise diminishes the risk of SCD during vigorous exertion
Whang (2006)^54^	USA	Prospective, nested case-crossover study	Self-reported	Sex = Females Sample = 69 693 Events = 157	~18.0	HR (95% CI) of 0.51 (0.47-0.56) comparing ⩾4 h/week of moderate-to-vigorous exercise vs inactivity	Inverse association between PA and SCD
Chiuve (2011)^55^	USA	Prospective cohort	Self-reported	Sex = Females Sample = 81 722 Events = 321	26.0	RR (95% CI) of 0.67 (0.50-0.90) for women exercising ⩾30 min/day and 0.47 (0.30-0.72) for those exercising ⩾6.0 vs ⩽1.0 h/week vs ⩽1 h/week	Inverse association between PA and SCD
Lahtinen (2012)^56^	Finland	IPD meta-analysis of 4 cohorts	Self-reported	Sex = Both Sample = 27 629 Events = 716	10.0	HR (95% CI) of 0.51 (0.43-0.62) comparing moderate-to-high LTPA with low LTPA	Inverse association between PA and SCD
Aune (2020)^39^	Multiple countries	Meta-analysis of 8 cohorts	Self-reported	Sex = Both Sample = 136 298 Events = 1193	6–26	RR (95% CI) of 0.52 (0.45-0.60) comparing high vs lowest PA levels; similar associations in men and women Dose-response analysis: RR (95% CI) of 0.68 (0.55–0.86) per 20 MET-hours/week.	Inverse association between PA and SCD
Li (2022)^60^	China	Retrospective cohort	Measured by implantable cardioverter defibrillators	Sex = Both Sample = 133 Events = 23	4.8	HR (95% CI) of 3.35 (1.39-8.03) comparing ⩽93 min/day of objectively measured PA vs those exceeding this threshold	Daily PA of about 1.5 h is associated with lower cardiac death risk in patients with age > 75 years
Qiu and Xing (2023)^61^	UK	Prospective cohort	Accelerometer-measured PA	Sex = Both Sample = 98 893 Events (cardiac arrest) = 282	7.3	The risk of cardiac arrest declined steeply with increasing PA volume, with the most notable risk reduction observed up to 360 min/week for moderate PA and 20 min/week for vigorous PA	Accelerometer-measured PA, particularly moderate and vigorous PA is associated with a lower cardiac arrest risk
Cardiorespiratory fitness							
Laukkanen (2010)^35^	Finland	Prospective cohort	Respiratory gas exchange analyzer	Sex = Males Sample = 2368 Events = 146	17.0	RR (95% CI) of 0.78 (0.71-0.84) per 1-MET increase in CRF	Inverse association between CRF and SCD
Jiménez-Pavón (2016)^63^	USA	Prospective cohort	Maximal treadmill testing	Sex = Both Sample = 55 456 Events = 109	~28.0	HR (95% CI) of 0.56 (0.35–0.90) and 0.52 (0.30-0.92) for moderate and high CRF compared with low CRF 0.86 (0.77-0.96) per 1-MET increase in CRF No significant differences in association by sex	Inverse association between CRF and SCD
Jiménez-Pavón (2019)^40^	Finland and USA	Meta-analysis of 2 cohorts	Respiratory gas exchange analyzer and maximal treadmill testing	Sex = Both Sample = 57,824 Events = 109	~17.0-28.0	HR (95% CI) of 0.81 (0.74-0.89) per 1-MET increase in CRF	Inverse association between CRF and SCD
Hernesniemi (2019)^64^	Finland	Prospective cohort	Maximal work level achieved during cycle ergometer testing	Sex = Both Sample = 2697 Events = 98	9.1	HR (95% CI) of 0.69 (0.52-0.93) per 1-standard deviation increase in CRF No significant differences in association by sex	Inverse association between CRF and SCD
Aune (2020)^39^	Finland and USA	Meta-analysis of 2 cohorts	Respiratory gas exchange analyzer and maximal treadmill testing	Sex = Both Sample = 57 824 Events = 255	22.0	RR (95% CI) of 0.58 (0.41-0.81) comparing the highest vs lowest CRF levels, and 0.49 (0.33-0.73) per 5-MET increase in CRF	Inverse association between CRF and SCD

Abbreviations: CI, confidence interval; CRF, cardiorespiratory fitness; HR, hazard ratio; LTPA, leisure-time physical activity; MET, metabolic equivalent; PA, physical activity; RR, relative risk; SCD, sudden cardiac death.

## Special Populations

While the protective benefits of PA and CRF for cardiovascular health are well established, questions remain regarding their effects in certain populations, particularly those engaged in high-intensity physical activity (HIPA) such as endurance athletes. The volumes and intensities of HIPA far exceed those recommended in clinical guidelines,^93^ prompting debate about whether there is an upper limit beyond which PA may become harmful, a concept referred to as the “Extreme Exercise Hypothesis.”^94^ This hypothesis suggests a U- or reverse J-shaped dose–response curve between PA and adverse cardiovascular outcomes, particularly SCD.^94^ In a 2011 systematic review, Dahabreh and Paulus reported that episodic PA was associated with a nearly fivefold increased risk of SCD (RR = 4.98, 95% CI: 1.47-16.91). However, habitual PA significantly attenuated this risk, with each additional session per week reducing the relative risk by 30%.^95^ In a retrospective analysis of marathon finishers, Roberts et al. (2013) reported low overall rates of SCA among both men (3.4 per 100 000) and women (0.6 per 100 000), highlighting the rarity of events despite the intensity of the activity.^96^ Similarly, Risgaard et al. (2014) found no difference in the incidence of sports-related SCD between competitive and noncompetitive athletes, suggesting that competition status alone is not a key determinant of risk.^97^ A Finnish autopsy study by Toukola et al. (2015) identified male sex, ischemic heart disease, cardiac hypertrophy, and myocardial scarring as common underlying pathologies in exercise-related SCD cases.^98^ Consistent with this, a 2016 review by Goodman et al. showed that acute exercise increases the risk of cardiovascular events, especially with advancing age and vigorous intensity, but the absolute risk remained extremely low (<0.01 per 10 000 participant-hours) and was markedly lower in individuals with long-standing PA.^99^ In a Swedish national study, Wisten et al. (2019) found an increased risk of exercise-related SCD in young males with inherited conditions such as hypertrophic cardiomyopathy (HCM) and arrhythmogenic right ventricular cardiomyopathy (ARVC).^100^ In contrast, a prospective cohort of over 50 000 male participants in a long-distance cross-country ski event showed no association between race frequency or finishing time and the risk of ventricular arrhythmias or cardiac arrest.^101^ In 2020, Tulppo et al. reported a U-shaped association between leisure-time PA and SCD in patients with CAD. Inactive patients had an elevated SCD risk (HR = 2.45, 95% CI: 1.01-5.98), but symptomatic individuals who were highly active also showed increased risk (HR = 7.46, 95% CI: 2.32-23.9), compared with those engaging in moderate PA.^102^ Ma et al. (2022) analyzed 374 sports-related SCDs in China and found that events occurred most frequently in males aged 39 to 59, with basketball and running being the most common activities. Most SCDs occurred during or within 1 hour of exercise.^103^ In a large systematic review and meta-analysis, Lear et al. (2022) found that the global incidence of SCD among athletes remained low, with <2 events per 100,000 athlete-years, based on data from 12 studies with low risk of bias.^104^ Bohm et al. (2023) further clarified this risk in a prospective cohort of young adults (aged 18–35) in Germany and Paris. Among 147 cases of sports-related SCA (95.2% male), only 12 (8.2%) occurred in young competitive athletes. Coronary artery disease was the leading cause, highlighting its relevance even in younger adults.^105^ In 2025, Lee et al. studied 75 SCD cases in patients with HCM and found that 20% of events occurred during high-intensity PA (⩾6 METs). Younger age was the only independent predictor of SCD during high-intensity activity, emphasizing the vulnerability of certain subgroups.^106^ Beyond cardiac structure, metabolic and systemic risk factors also play a role. In a 2021 review, Niebauer and Burtscher found that prior myocardial infarction and hypertension were major SCD risk factors in skiers, while hypercholesterolemia and diabetes were more relevant for mountain hikers. Importantly, regular high-intensity exercise was protective in both groups (e.g., OR = 0.17 for skiers).^107^ With growing interest in extreme endurance, the “Extreme Exercise Hypothesis” has attracted attention. Although several studies support its plausibility—particularly the association between very high CRF levels (>10 METs) and increased risk of atrial fibrillation (AF) ^108^—no study has reported a direct link between high CRF and increased SCD risk. As such, the current body of evidence suggests that while AF risk may rise with very high fitness, the association between high CRF and SCD remains protective.

The available literature indicates that sports- and exercise-related SCD is rare, particularly among habitually active individuals. However, risk varies by age, sex, fitness level, and underlying cardiovascular conditions. Young athletes are more likely to experience SCD due to inherited conditions, while ischemic disease predominates in older adults. The acute risk of SCD is highest in sedentary individuals engaging in episodic vigorous exercise, emphasizing the importance of progressive training and screening in at-risk populations. Despite speculation around the “Extreme Exercise Hypothesis,” the evidence does not currently support avoiding high-intensity exercise in trained individuals. On balance, the benefits of sustained PA and CRF far outweigh the risks, especially when activity is approached in a gradual and informed manner.

## Synergistic or Joint Effects: PA, CRF, and Combined Risk Factor Modification in SCD Prevention

Evidence from numerous epidemiological studies suggests that CRF is not only a powerful predictor of cardiovascular outcomes but may also be among the strongest modifiable risk factors for SCD. The protective effect of high CRF levels has been shown to attenuate, and in some cases negate, the increased risk of SCD associated with several traditional and emerging cardiovascular risk factors.^109–122^ Moreover, CRF has also been found to potentiate the beneficial effects of protective lifestyle behaviors, such as sauna bathing, further reinforcing its role as a central modifier in the SCD risk landscape.^123–125^

In one of the earlier investigations of joint effects, Hagnas et al. (2017) examined the interplay between exercise-induced ST-segment depression and CRF in the KIHD cohort. They found that elevated CRF levels (⩾8 METs) significantly attenuated the increased risk of SCD associated with exercise-induced ST depression, highlighting a potential protective effect of CRF in the presence of latent ischemia.^126^ In 2018, Hagnas et al. further demonstrated that low LTPA and low CRF were independently associated with increased SCD risk, and their combined presence conferred a substantially higher risk than either factor alone (HR = 2.2, 95% CI: 1.4-3.3).^127^ This underscores the synergistic protective role of both habitual PA and fitness. In a related study, Laukkanen et al. (2018) investigated the joint effects of CRF and frequency of sauna bathing (FSB) and found that while both factors individually reduced the risk of SCD, their combination offered the greatest benefit. Men with both high CRF and high FSB had a 69% lower risk of SCD (HR = 0.31, 95% CI: 0.16-0.63) compared to those with low CRF and low FSB, suggesting a powerful additive or even synergistic effect.^124^ Also in 2018, Jae et al. evaluated the interaction between BMI and CRF. They found that obesity-related increases in SCD risk were significantly attenuated among fit men, with obese-unfit men experiencing a substantially higher risk (HR = 1.80, 95% CI: 1.06-3.06) compared to obese-fit men (HR = 1.22, 95% CI: 0.66-2.25).^128^ A subsequent study by Kunutsor et al. (2023) provided further support, reporting modest evidence of additive and multiplicative interactions between obesity and low CRF in relation to SCD.^129^ In a 2021 analysis of the KIHD cohort, Jae et al. demonstrated that the association between low socioeconomic status (SES) and SCD was significantly modified by CRF, with higher CRF attenuating the increased risk of SCD in men with low SES (HR = 1.41, 95% CI: 0.92-2.16 for low SES and fit men vs HR = 2.04, 95% CI: 1.37-3.02 for low SES and unfit men).^122^ Laukkanen et al. (2022) examined the combined effects of CRF and inflammation, measured by high-sensitivity C-reactive protein (hsCRP), and reported that high CRF levels counteracted the elevated SCD risk associated with systemic inflammation. Notably, there was evidence of positive additive and multiplicative interactions between hsCRP and CRF (HR = 1.72, 95% CI: 1.10-2.69 for high hsCRP and low CRF vs HR = 0.86, 95% CI: 0.39-1.88 for high hsCRP and high CRF).^130^ In 2023, Laukkanen et al. again used the KIHD cohort to explore the interaction between systolic blood pressure (SBP) and CRF. They found that medium-to-high CRF levels offset the elevated risk of SCD associated with high SBP, with only modest risk in those with high SBP and high CRF (HR = 1.38, 95% CI: 0.84-2.26), compared to those with high SBP and low CRF (HR = 2.67, 95% CI: 1.76-4.05).^131^ Most recently, Kunutsor et al. (2024) evaluated the interaction between T2D and CRF. Their findings demonstrated that medium-to-high CRF levels substantially attenuated the increased risk of SCD in men with T2D, and again, evidence of additive and multiplicative interactions was observed (HR = 3.34, 95% CI: 2.00-5.57 for T2D and low CRF vs HR = 1.46, 95% CI: 0.46-4.65 for T2D and medium-high CRF).^132^

While strong and consistent evidence supports the modifying role of CRF, findings on whether PA alone modifies the effects of other risk factors on SCD risk have been more mixed. Several studies have reported inconsistent or null findings regarding the capacity of higher PA levels to mitigate the adverse effects of traditional cardiovascular risk factors on SCD or related outcomes.^133–135^ This discrepancy may stem from variability in how PA is measured (e.g., self-report vs accelerometry), differences in population characteristics, or a true lesser effect compared to CRF.

Collectively, the evidence suggests that CRF plays a critical modifying role in the relationship between numerous cardiovascular risk factors and the risk of SCD. High CRF levels have been shown to attenuate or even neutralize the harmful effects of obesity, hypertension, systemic inflammation, diabetes, socioeconomic disadvantage, and other physiological stressors. Moreover, CRF can enhance the effects of health-promoting behaviors such as sauna bathing. Although less consistent, the synergistic potential of PA when combined with other risk factor modifications warrants further investigation.

## Challenges and Limitations in the Literature

Despite a growing body of evidence supporting the inverse associations between PA, CRF, and the risk of SCD, several important limitations in the current literature restrict our ability to draw definitive conclusions or translate findings into precise clinical guidance. One of the foremost limitations is the lack of RCTs specifically targeting SCD as an endpoint. While numerous RCTs have demonstrated the cardioprotective benefits of PA and exercise training for a variety of cardiovascular outcomes, very few have been powered or designed to assess the impact of these interventions on SCD specifically. Given the relatively low incidence of SCD in the general population, especially among asymptomatic individuals, trials would require large sample sizes and long-term follow-up, which may pose logistical and financial challenges. Additionally, although observational studies consistently report protective associations between higher levels of PA and CRF and lower risk of SCD, they are inherently limited by residual confounding and potential reverse causality. To address this, MR offers a promising approach, but to date, no MR studies have been conducted to assess the causal effects of PA or CRF on SCD. This represents a significant gap in the literature, particularly given the growing availability of large-scale genetic datasets with adjudicated cardiovascular outcomes. The lack of MR studies may, in part, be due to the complexity of identifying valid genetic instruments for PA and CRF and the rarity of well-phenotyped SCD endpoints in biobanks and cohort studies. Another major limitation is the scarcity of studies that have comprehensively characterized dose–response relationships between PA or CRF and SCD risk. While some evidence suggests a graded inverse association, few studies have quantified the exact thresholds or intensity levels of PA or CRF that yield maximal protection. Understanding whether there are plateaus, minimal effective doses, or upper safety limits is essential for informing public health recommendations and individualized exercise prescriptions. Moreover, current evidence often lacks granularity regarding type, frequency, and intensity of PA, which are key to understanding differential effects on SCD risk. Beyond these issues, heterogeneity in measurement methods further complicates interpretation across studies. PA is commonly assessed through self-reported questionnaires, which are subject to recall and social desirability biases. Similarly, while CRF is ideally measured using CPX, many large epidemiological studies rely on estimated CRF derived from indirect methods or prediction equations,^25,49,50^ which may not capture true physiological fitness and can vary in accuracy across populations.^25,26^ The literature is also disproportionately composed of studies from Western, predominantly male cohorts, limiting the generalizability of findings to women, ethnic minorities, and other global populations. Additionally, few studies have explored age-specific or sex-specific thresholds of PA or CRF required for optimal risk reduction, despite known biological differences in cardiovascular physiology and event presentation. Furthermore, while many studies explore individual associations of PA or CRF with SCD risk, fewer investigate joint or interactive effects with other cardiovascular risk factors in a statistically robust manner. Although recent work suggests that CRF may mitigate the adverse effects of factors such as obesity, hypertension, and systemic inflammation, more research is needed to understand the complex interplay between PA, fitness, lifestyle, and risk factor profiles in relation to SCD. Finally, from a causal inference standpoint, no MR studies have yet evaluated the causal effects of PA or CRF on SCD. The absence of such studies represents a critical gap in the literature. One contributing challenge is the difficulty in identifying genetic variants that reliably and specifically capture CRF, which is a complex phenotype influenced by both genetic and environmental factors. While recent GWAS have begun to identify candidate loci for PA and CRF, these instruments are still evolving and may lack the precision required to serve as robust proxies for MR analyses targeting SCD.

## Clinical and Public Health Implications

The cumulative evidence from this review reinforces the substantial and consistent protective effects of regular PA and higher CRF against the risk of SCD. These findings carry significant clinical and public health implications and support the prioritization of PA and CRF enhancement as central strategies in SCD prevention. From a clinical perspective, both PA and CRF have demonstrated strong inverse and dose-dependent relationships with SCD risk. The optimal protective effect of PA was observed at levels consistent with current public health recommendations—150 to 300 minutes of moderate-intensity or 75 to 150 minutes of vigorous-intensity activity per week, or a combination thereof, alongside at least two days of strength training.^93^ A summary of the key PA and CRF thresholds associated with SCD risk reduction is presented in Table 2. Despite this alignment with existing guidelines, a substantial proportion of the population fails to meet these thresholds. Notably, gender and racial disparities persist, with lower PA levels particularly observed in younger and older women and non-Hispanic African American women.^136^ These findings underscore the need for targeted outreach and culturally tailored strategies to enhance PA uptake in underrepresented and higher-risk groups.

**Table 2.** Summary of physical activity and cardiorespiratory fitness thresholds associated with reduced sudden cardiac death risk.

Measure	Threshold/level	Associated risk reduction	Notes/recommendations
Moderate-Intensity PA	150–300 min/week	40–50% reduction in SCD risk	Based on public health guidelines; dose-response relationship observed—greatest risk reduction occurs up to 360 min/week of moderate PA, beyond which benefits tend to plateau
Vigorous-Intensity PA	75–150 min/week	Similar reduction as moderate PA	Greatest risk reduction occurs up to 20 min/week of vigorous PA, beyond which benefits tend to plateau; greater CRF gains observed with vigorous activity
CRF (in METs)	⩾8–10 METs	>40–50% reduction in SCD risk	Strong inverse association; ideal clinical target
Exercise Intensity	Higher intensity leads to greater CRF improvement	Indirectly reduces SCD risk	High-intensity exercise safe for most individuals without CVD
Strength Training	⩾2 days/week	Supports PA guidelines and CRF	Complements aerobic exercise in enhancing fitness

Abbreviations: CRF, cardiorespiratory fitness; CVD, cardiovascular disease; METs, metabolic equivalents; PA, physical activity; SCD, sudden cardiac death.

CRF has emerged as a particularly powerful predictor of SCD, with individuals achieving ⩾8 to 10 METs experiencing risk reductions of over 40% to 50%. This evidence suggests that CRF thresholds could inform clinical risk stratification and guideline development, providing an objective and modifiable target for SCD prevention. Importantly, while CRF is partly influenced by genetic predisposition, the majority of an individual’s CRF level is determined by habitual PA and structured exercise training.^137^ In particular, vigorous or high-intensity PA has been shown to produce the greatest improvements in CRF,^138,139^ highlighting the importance of exercise intensity in optimizing cardiovascular protection.

In terms of clinical application, a critical distinction exists between directly measured CRF (typically via CPX) and estimated CRF derived from predictive algorithms using submaximal tests or self-reported data. While estimated CRF offers practicality and scalability, especially in large population studies and clinical settings with limited resources, it lacks the precision and physiological accuracy of direct measurements. This discrepancy can impact clinical decision-making, as under- or overestimation of fitness levels may affect individual risk stratification, guideline adherence, and exercise prescription. Moreover, variation in estimation methods across studies can hinder direct comparability of findings, potentially leading to inconsistent conclusions regarding CRF thresholds and their relationship with SCD. Standardization of estimation protocols and greater adoption of CPX where feasible may help bridge these gaps and enhance the translational utility of CRF in preventive cardiology.

Routine assessment of CRF in clinical practice, using standardized protocols or validated estimation methods, should be prioritized, not only as a marker of physical capacity but as a powerful clinical vital sign associated with long-term mortality and SCD risk. This is particularly relevant for some population groups such as African American, who have been shown to exhibit lower baseline CRF and more limited gains in CRF following structured exercise training programs, compared to White individuals.^140^ Such disparities highlight the potential value of individualized and adaptive training regimens to support equitable cardiovascular outcomes. In older adults, maintaining CRF is especially important, as CRF levels typically decline with aging, comorbid conditions, and reductions in PA participation.^141^ Given the heightened SCD risk in older populations and the observed attenuation of SCD risk with higher CRF, strategies aimed at preserving or enhancing CRF in later life should be central to clinical and community-based preventive efforts.

Importantly, much of the current evidence base stems from studies conducted predominantly in Western populations, particularly among middle-aged and older White males. This demographic skew limits the generalizability of findings to women, younger individuals, and racially or ethnically diverse populations. It also presents a barrier to advancing health equity, as underrepresented groups may exhibit differing baseline levels of PA and CRF, unique sociocultural barriers to engagement, and distinct pathophysiological risk profiles. Without adequate representation in research, opportunities to tailor prevention strategies and reduce disparities in SCD outcomes remain constrained.

While the health benefits of PA and exercise are well-established, their safety profile, particularly in the context of vigorous or high-volume activity, warrants careful consideration. Although concerns have been raised about potential risks, especially among endurance athletes or individuals with underlying cardiovascular conditions, the absolute risk of exercise-induced SCD is exceedingly low in those who are regularly active and free of significant heart disease. Nonetheless, transient elevations in SCD risk can occur during acute bouts of vigorous exertion, particularly in sedentary or high-risk individuals who are unaccustomed to such activity. To minimize these risks and promote safe participation, leading guideline bodies such as the American College of Sports Medicine (ACSM) and the American Heart Association (AHA) recommend pre-participation screening to assess cardiovascular risk, especially before initiating high-intensity exercise.^142,143^ Key elements include evaluation of medical history, current activity level, and symptoms, followed by individualized, progressive exercise programs. Medical clearance is advised for sedentary individuals and those with established CVD prior to engaging in vigorous activity.^142^ Gradual increases in exercise intensity and volume, along with patient education and ongoing clinical oversight, are essential to ensure safety while maximizing the cardioprotective effects of PA.

Emerging digital health technologies, including wearable devices and smartphone-based tools capable of estimating CRF and tracking PA in real-time, hold promise for enhancing both clinical risk assessment and public health surveillance. These technologies can facilitate continuous monitoring, early detection of abnormal cardiovascular responses, and personalized feedback to support behavior change. When appropriately validated, they may also offer scalable solutions for screening, stratification, and targeted intervention in populations at risk for SCD.

In conclusion, these findings call for more systematic integration of PA promotion and CRF assessment into routine preventive care, risk prediction, and health policy. Strengthening these efforts has the potential to reduce the burden of SCD, enhance population health, and address long-standing disparities in cardiovascular outcomes.

## Interventions for Improving Levels and Uptake of PA and Levels of CRF

Given the robust evidence linking both PA and CRF to a significantly reduced risk of SCD, implementing effective interventions to improve PA uptake and CRF levels represents a vital public health and clinical priority. Despite the widespread recognition of their health benefits, global levels of PA remain suboptimal,^144^ necessitating urgent and coordinated strategies to promote movement across the population.

Recognizing the strong association between insufficient PA and major non-communicable diseases (NCDs), World Health Organization (WHO) member states are committed to achieving a 10% relative reduction in physical inactivity by 2025 as part of the global NCD prevention strategy.^145^ PA in any form—whether through leisure, transport, work, or domestic activities—has well-documented health benefits, including cardiovascular protection. However, data indicate that a substantial proportion of adults worldwide do not meet recommended levels, with notable disparities across sex, age, and ethnicity.^146,147^. To address these gaps, a multisectoral approach involving health, urban planning, education, and transportation sectors is essential. Recommended strategies for increasing population PA have included ^144^ (i) improving infrastructure for non-motorized transportation such as walking and cycling, and promoting their safe use; (ii) encouraging active leisure time activities through structured programs, workplace initiatives, and school-based interventions; (iii) expanding access to public open spaces, parks, and recreational facilities that enable safe, convenient PA for all age groups; (iv) addressing cultural and social barriers, particularly among women, to facilitate inclusive PA participation; and (v) tailoring interventions to groups with historically lower PA levels, including younger and older women and non-Hispanic African American women, who consistently report the lowest PA engagement in population-based studies.^136^ Healthcare providers play a central role in promoting PA by incorporating PA counselling into routine care, using tools such as the Physical Activity Vital Sign (PAVS), and connecting patients to community-based PA programs.^148,149^

CRF, a powerful and independent predictor of cardiovascular and all-cause mortality, is largely influenced by habitual PA and structured exercise training. Although the exact amount of PA required to achieve specific CRF thresholds varies, moderate-intensity exercise consistent with PA guidelines typically enables middle-aged individuals to reach moderate CRF levels (>8 METs) ^138,139^—levels associated with substantial protection against SCD. Based on large-scale cohort data, optimal CRF levels (age-standardized METs: ~9 in men, ~7 in women) approximate 130 to 148 minutes/week of brisk walking, equating to ~8–9 MET-hours/week.^150^ Additionally, an exercise capacity above 5 METs can be achieved by regularly performing activities exceeding 3 METs, such as moderate-to-vigorous PA.^151^ Despite these general recommendations, interindividual variability in CRF response to exercise exists and should be accounted for when designing fitness programs.

Beyond exercise, complementary interventions may enhance CRF gains and these include (i) nutrition therapy, including dietary modifications such as the DASH or Mediterranean diets, and supplementation with amino acids, n-3 polyunsaturated fatty acids, or L-carnosine; these may support improvements in CRF, though current evidence remains limited;^152^ (ii) pharmacologic therapies, particularly in populations with low baseline CRF (e.g., heart failure), may hold promise. Medications such as renin–angiotensin–aldosterone system inhibitors, hydralazine, and digoxin have been associated with improvements in VO₂peak ^153,154^ and warrant further investigation in broader populations; and (iii) addressing modifiable lifestyle factors that strongly influence CRF is also essential. For instance, smoking cessation has been shown to significantly reduce cardiovascular damage and improve CRF within months.^155^ Body composition management, particularly reducing fat mass and increasing lean muscle mass, directly enhances CRF.^156^ Weight loss, achieved through dietary changes and consistent PA, reduces cardiac workload and improves heart function, facilitating measurable improvements in CRF.^157^ Given the importance of CRF as both a marker and modifier of SCD risk, routine CRF assessment in clinical practice—using either direct CPX or validated non-exercise algorithms—should be considered essential. This is particularly relevant for older adults, in whom CRF levels decline with age, and for African American populations, who often present with lower CRF and more modest gains following standard interventions.

## Future Research Directions

**Figure 2.**
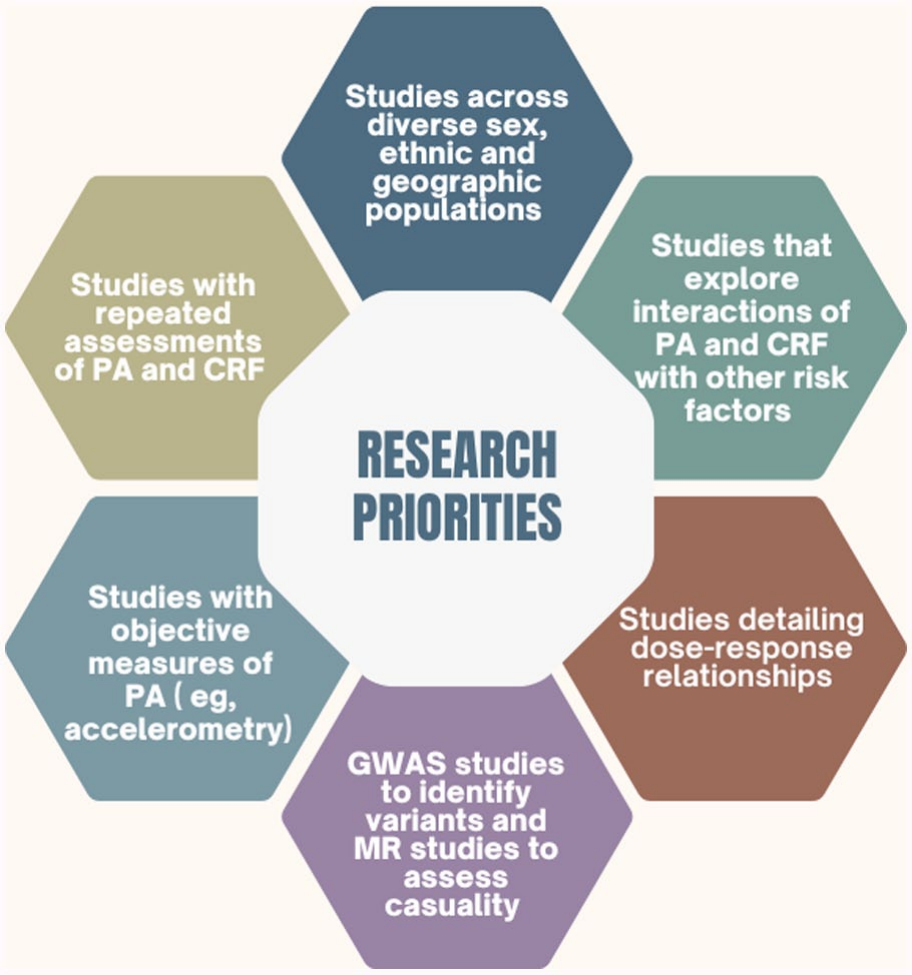
Research Priorities. Abbreviations: CRF, cardiorespiratory fitness; GWAS, genome-wide association studies; PA, physical activity.

While the current body of evidence strongly supports an inverse association between PA, CRF, and the risk of SCD, several important gaps remain that limit our understanding of causality, generalizability, and clinical applicability. Addressing these gaps through methodologically robust and diverse research will be essential to refine preventive strategies and ensure equitable cardiovascular health outcomes (Figure 2). A key methodological limitation of most existing studies is the reliance on single baseline measurements of PA and CRF, without accounting for their changes over time. This approach ignores within-person variability and introduces regression dilution bias, likely resulting in underestimation of the true strength of association. For example, reproducibility studies from the KIHD cohort demonstrated a regression dilution ratio for CRF of 0.58,^27,30,158,159^ indicating substantial variability across time within individuals. Importantly, longitudinal assessments of CRF can better capture behaviorally induced changes and reduce the confounding effects of genetic predisposition, as CRF changes are predominantly influenced by PA and sedentary behaviors rather than fixed genetic factors.^160^ Future cohort studies should prioritize repeated measurements of PA and CRF across follow-up periods to more accurately quantify their associations with SCD and understand temporal patterns of risk. Furthermore, although dose–response relationships have been described in some studies, there remains a lack of granular data to establish precise and personalized PA and CRF thresholds for SCD prevention. Future research should focus on detailed dose-response analyses that explore optimal levels of PA and CRF stratified by age, sex, and baseline risk, to inform more tailored public health recommendations. This is particularly important for populations such as older adults and women, who may differ in physiological response and baseline fitness levels compared to younger, predominantly male cohorts. To facilitate MR research, there is a pressing need for large-scale GWAS to identify robust genetic instruments for CRF and objectively measured PA phenotypes. Currently, genetic variants explaining sufficient variance in these complex traits remain limited. Developing precise and validated instruments is particularly challenging for CRF, which is a multifactorial trait shaped by both intrinsic (genetic) and extrinsic (behavioral and environmental) factors. Once robust instruments are available, MR can be employed to disentangle causal relationships between PA/CRF and SCD, helping to overcome biases such as confounding and reverse causality inherent in traditional observational studies. Another limitation of the current literature is the widespread reliance on self-reported PA measures, which are vulnerable to recall error and social desirability bias. As PA levels are often overestimated using self-report tools, the associations with outcomes such as SCD may be attenuated. Future studies should place greater emphasis on objective assessment methods, such as accelerometry, which provide more accurate and reliable estimates of movement behaviors and intensity. Leveraging wearable technologies and integrating such data into large prospective cohorts could substantially improve exposure classification and the strength of observed associations. There is also a clear need to broaden the diversity of study populations. Much of the evidence base stems from Western, predominantly male cohorts, limiting the generalizability of findings to women, racial/ethnic minorities, and low- and middle-income country populations. Ensuring representation across sex, ethnicity, SES, and geographic regions is essential to develop inclusive guidelines and prevent exacerbation of health disparities. Lastly, while many studies have assessed the independent associations of PA or CRF with SCD, few have comprehensively examined their joint or interactive effects with other cardiovascular risk factors (e.g., obesity, diabetes, inflammation, blood pressure) using statistically robust approaches. Future studies should aim to explore these interactions using additive and multiplicative models, which may reveal synergistic effects and help prioritize interventions in high-risk subgroups.

## Conclusions

This review underscores the compelling and consistent evidence that both PA and CRF are strong, modifiable risk indicators of SCD. Higher levels of PA and CRF are associated with substantial risk reductions, with optimal protection observed at moderate-to-vigorous PA levels and CRF thresholds of ⩾8–10 METs. Importantly, CRF not only confers independent protection but also attenuates the adverse effects of multiple cardiovascular risk factors. These findings highlight critical clinical and public health implications, emphasizing the need to prioritize the promotion of regular PA, the assessment of CRF in routine care, and the development of strategies to improve fitness levels across diverse populations. As SCD remains a devastating and often unpredictable event, integrating PA and CRF into preventive frameworks represents a vital opportunity for risk reduction. Looking ahead, future research should focus on longitudinal assessments, dose-response precision, objective PA measurement, causal inference using MR, and diversity in study populations. Addressing these gaps will be essential to optimize prevention strategies, refine clinical guidelines, and ensure that the benefits of PA and CRF are equitably realized across all segments of the population.
